# Supplementary material for: Influence of Increased Radiation Background on Antioxidative Responses of Helianthus tuberosus L
Source: Antioxidants (Basel). 2023 Apr 18;12(4):956. doi: 10.3390/antiox12040956 (PMC10135547; doi:10.3390/antiox12040956)
Supplement: Supplementary file 1 [file antioxidants-12-00956-s001.zip › antioxidants-2277470-supplementary.pdf]

antioxidants

Supplementary Materials

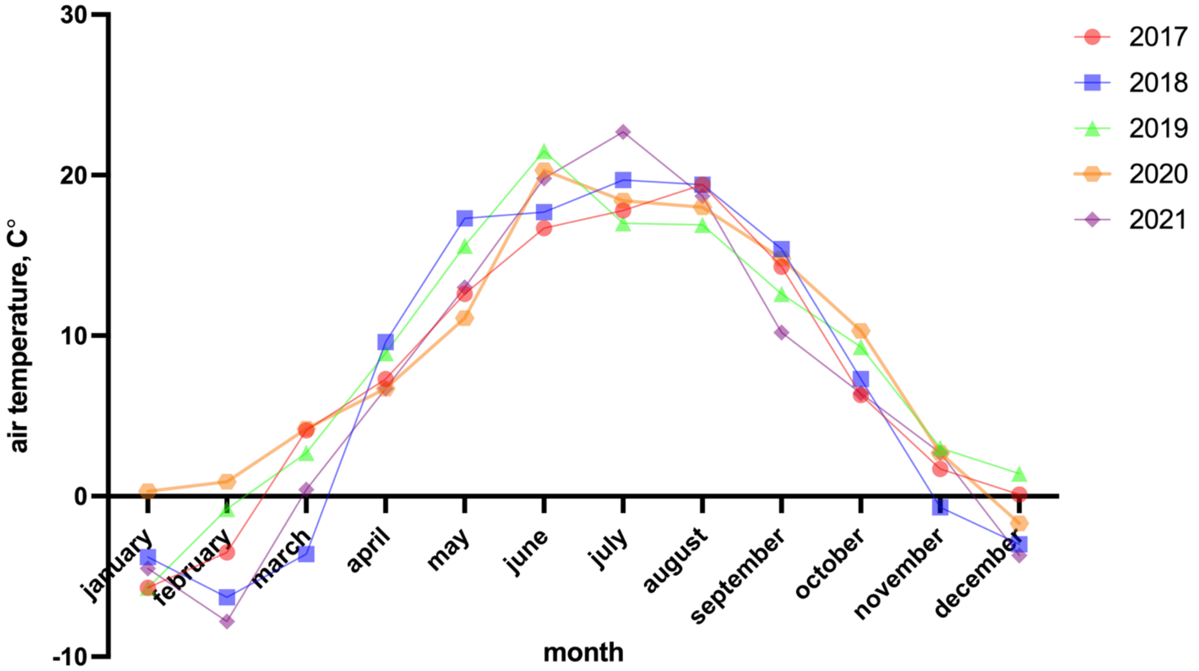

Figure S1. Variation of air temperature in polluted areas (OLKH and POZH). –

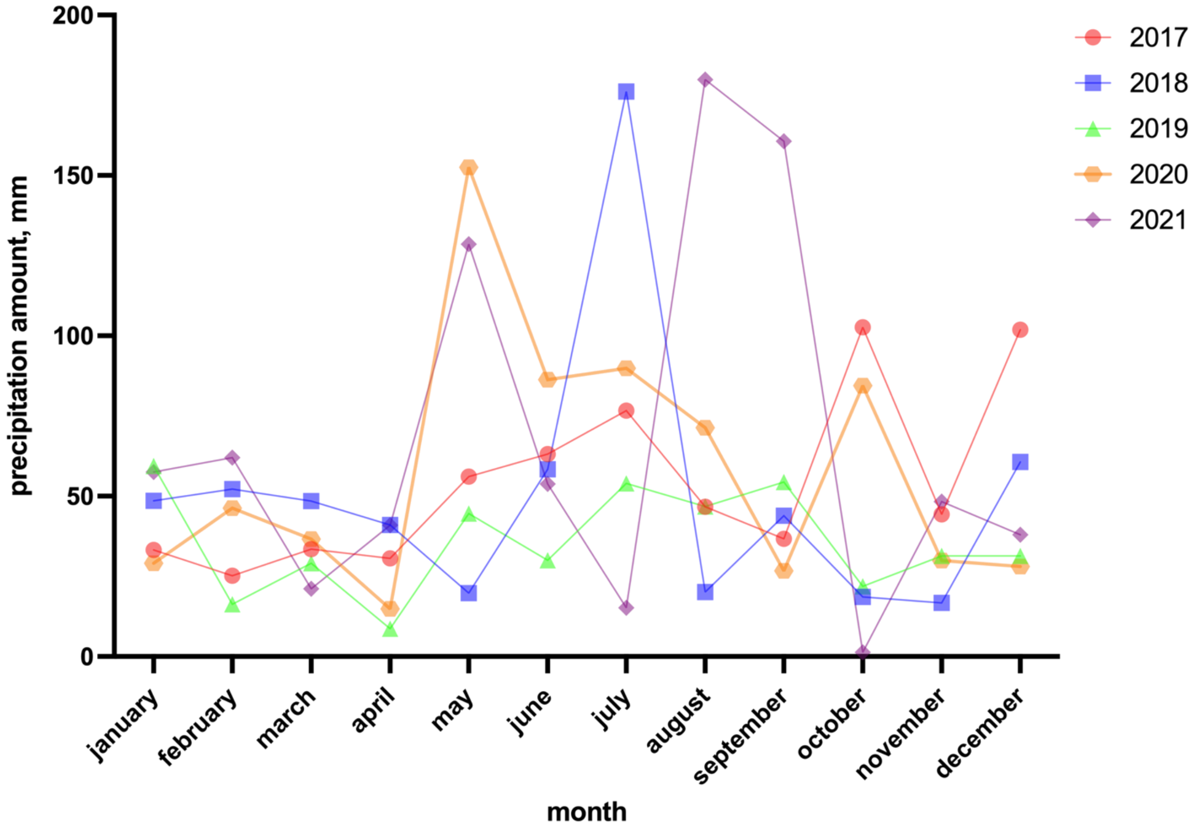

Figure S2. Variation in the amount of precipitation in polluted areas (OLKH and POZH).

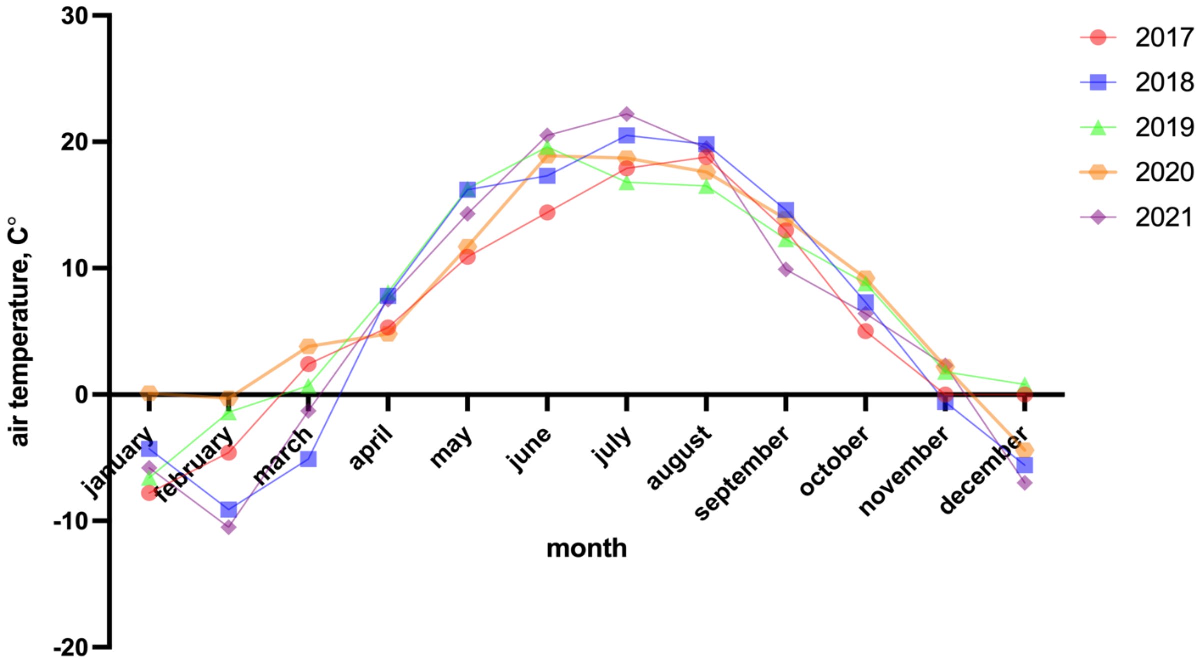

Figure S3. Variation of air temperature in pollution-free areas (K1 and K2).

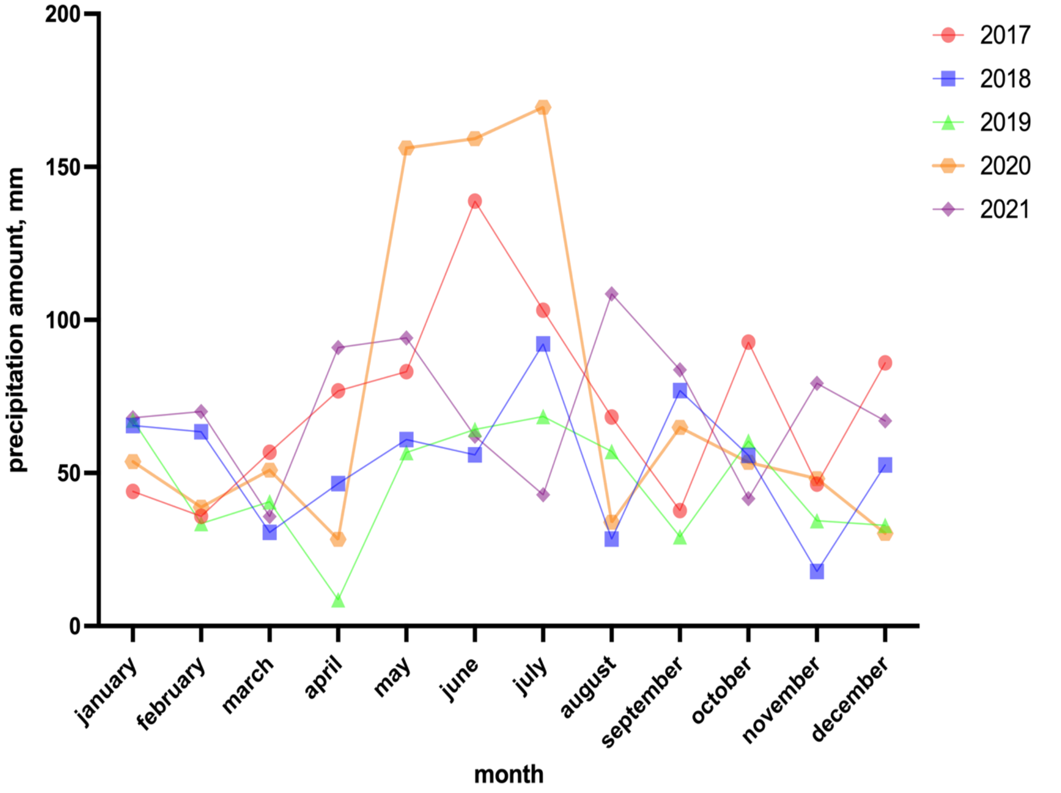

Figure S4. Variation in the amount of precipitation in pollution-free areas (K1 and K2).

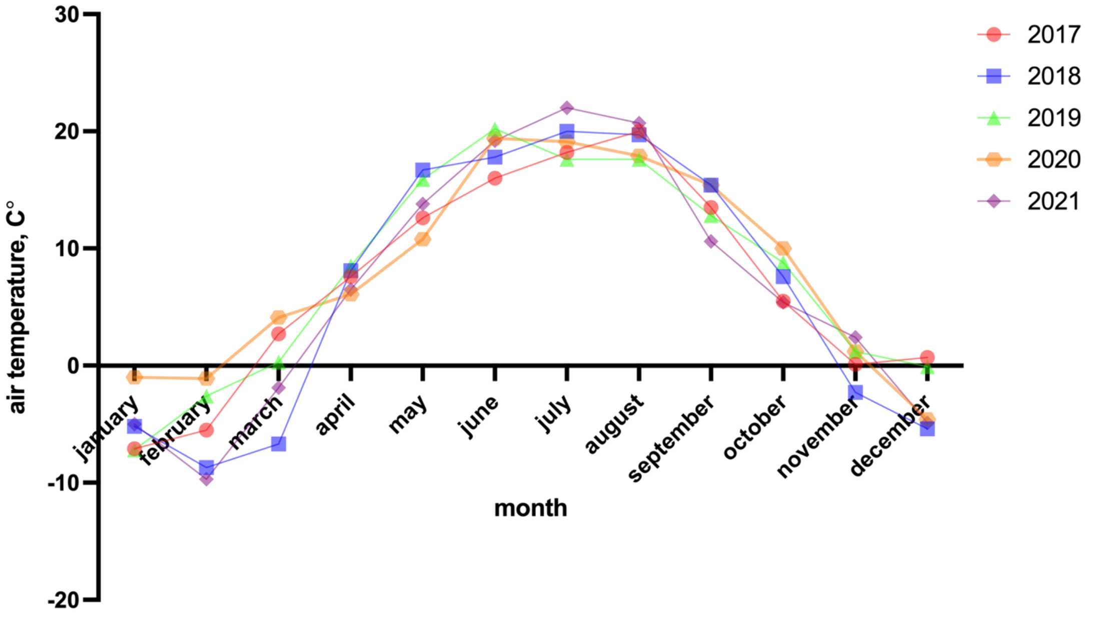

Figure S5. Variation of air temperature in pollution-free areas (K3).

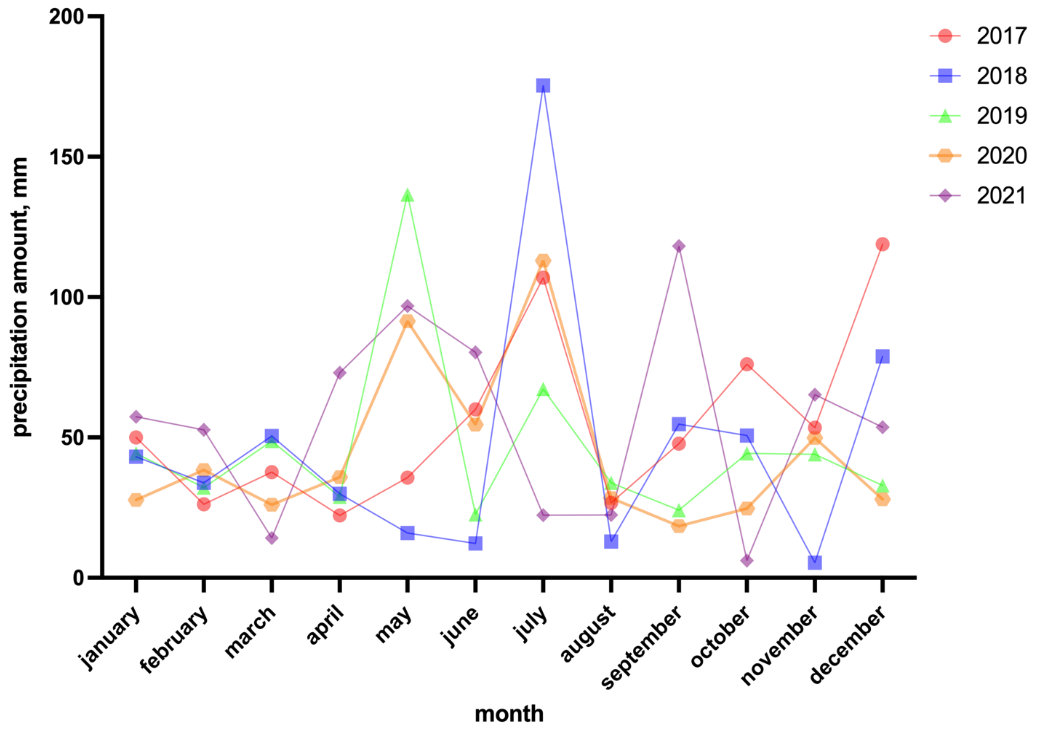

Figure S6. Variation in the amount of precipitation in pollution-free areas (K3).

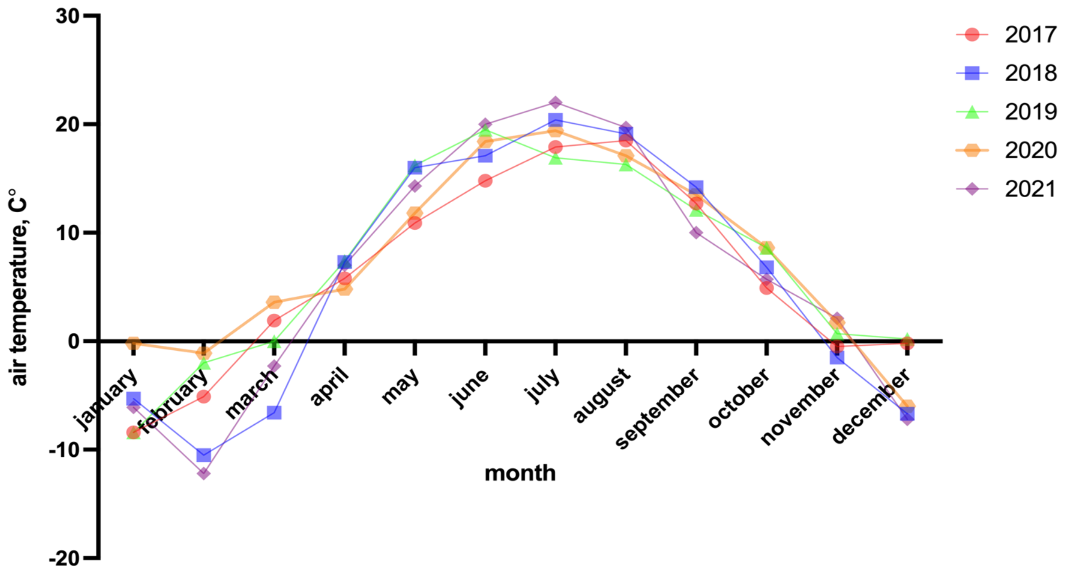

Figure S7. Variation of air temperature in pollution-free zones (K4).

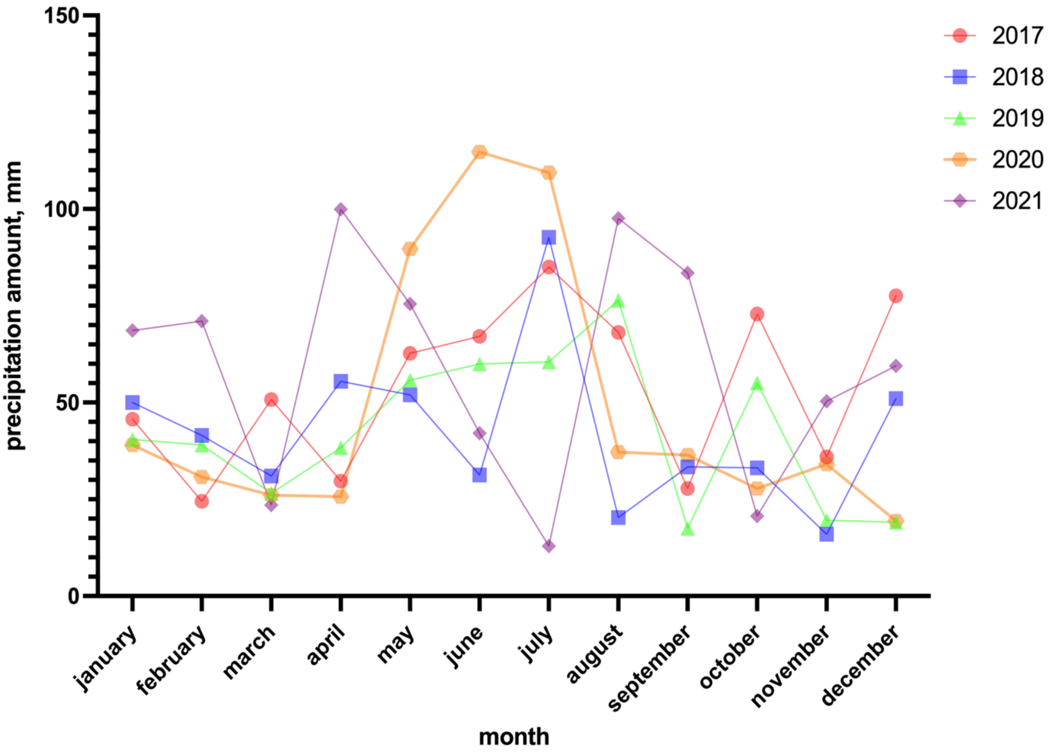

Figure S8. Variation in the amount of precipitation in pollution-free areas (K4).
